# Supplementary figures and images for: A Novel Cosegregating DCTN1 Splice Site Variant in a Family with Bipolar Disorder May Hold the Key to Understanding the Etiology
Source: Genes (Basel). 2020 Apr 18;11(4):446. doi: 10.3390/genes11040446 (PMC7231292; doi:10.3390/genes11040446)

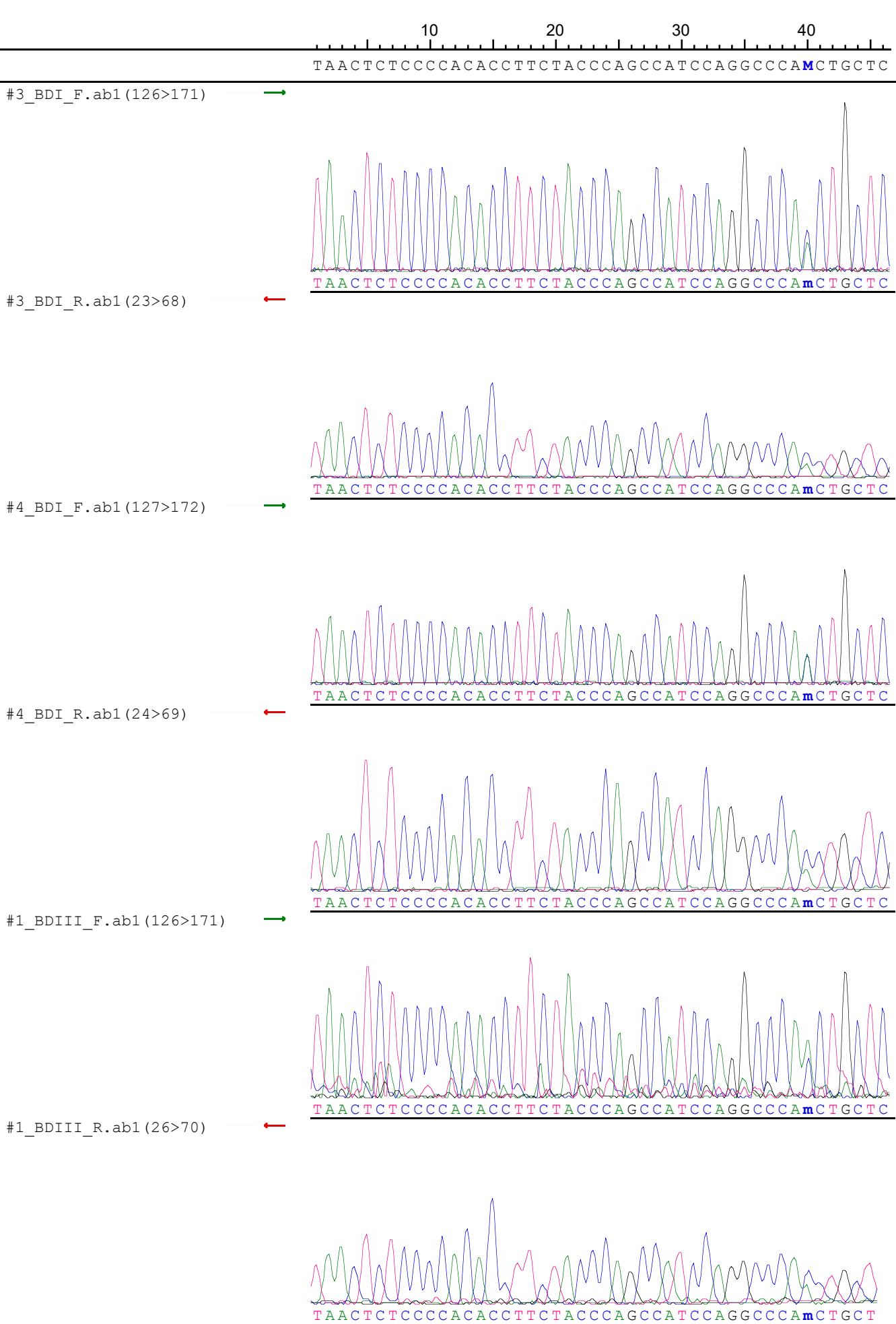

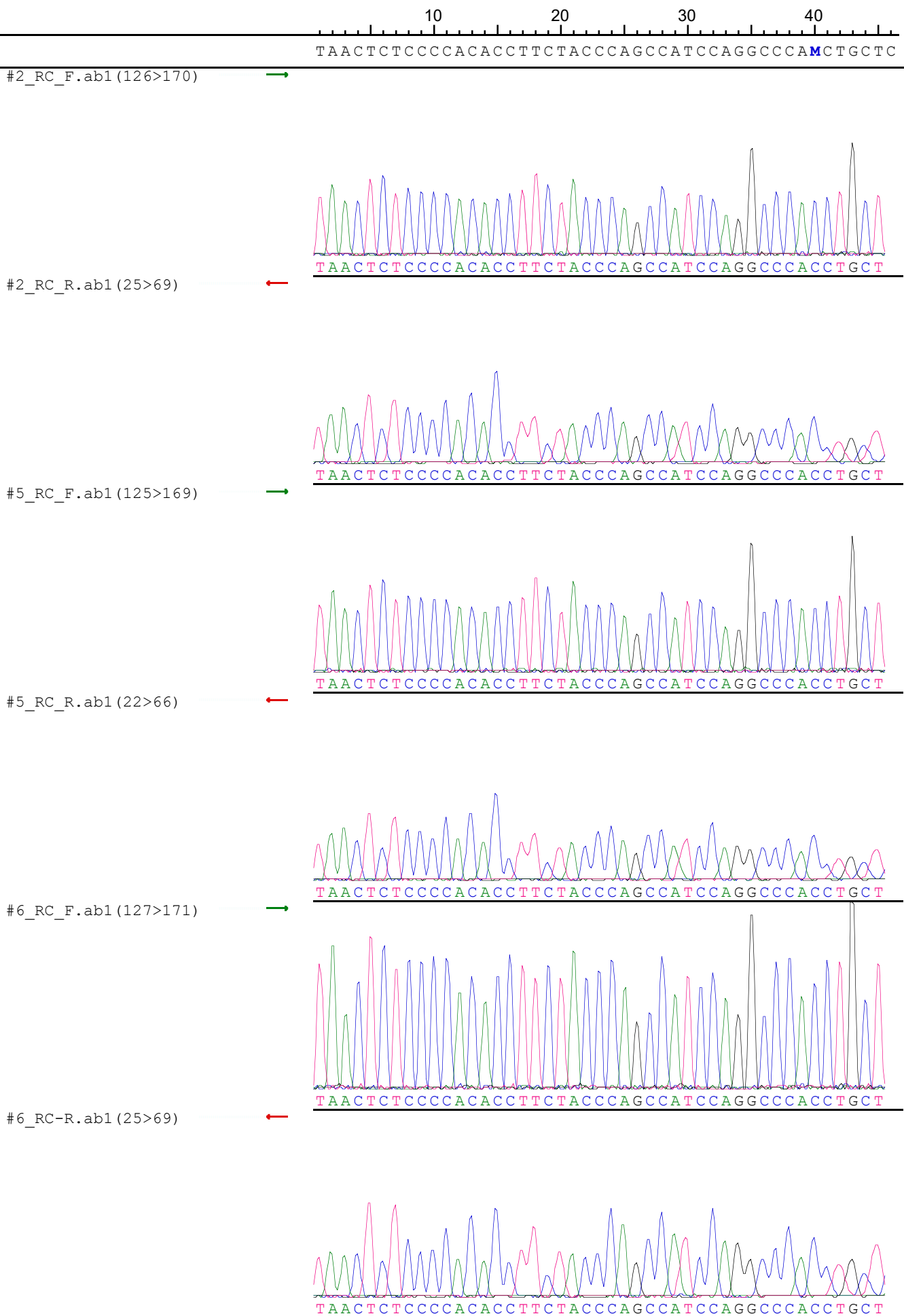

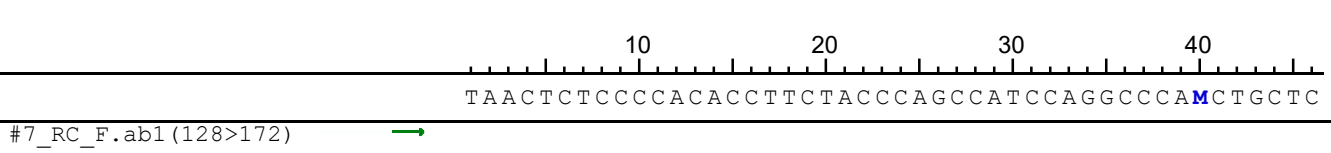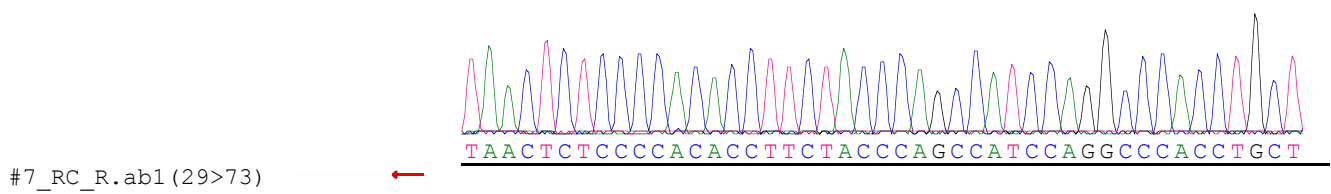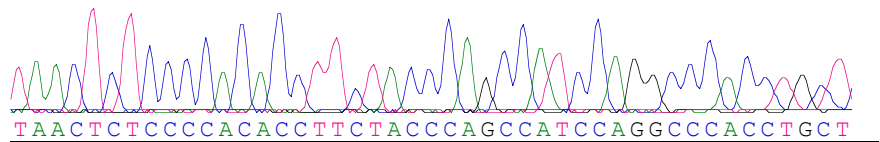

Supplement: Supplementary file 1 [file genes-11-00446-s001.zip › S2_Sanger_reads/Sanger alignment.pdf]
